# Supplementary material for: Translation and Cross-Cultural Adaptation of the International Questionnaire to Measure the Use of Complementary and Alternative Medicine (I-CAM-Q) for the Polish and Cross-Sectional Study
Source: Int J Environ Res Public Health. 2022 Dec 22;20(1):124. doi: 10.3390/ijerph20010124 (PMC9819037; doi:10.3390/ijerph20010124)
Supplement: Supplementary file 1 [file ijerph-20-00124-s001.zip › ijerph-1977583-supplementary.pdf]

International Journal of
Environmental Research and Public Health

MDPI

Supplementary Materials

Translation and Cross-Cultural Adaptation of the International Questionnaire to Measure the Use of Complementary and Alternative Medicine (I-CAM-Q) for the Polish and Cross-Sectional Study

Aneta Brygida Jędrzejewska, Barbara Janina Ślusarska, Krzysztof Jurek and Grzegorz Józef Nowicki

Table S1. The list of thematic groups on Facebook.

	Group name	Number of members (in thous.)
1.	CUKRZYCA ǀ Grupa mojacukrzyca.org	28.7
2.	CUKRZYCA 24H INFO	29.9
3.	DIABETYCY	10
4.	Jelita - to się leczy	3
5.	Wrzodziejące Zapalenia Jelit (Colitis Ulcerosa)	4.2
6.	Zdrowe jelita Zdrowi my - IBS, SIBO, Low Fodmap	9.7
7.	Zespół Jelita Drażliwego - nasze zmagania	10
8.	Jelita - Centrum Zdrowia	11
9.	Depresja, Stany Lękowe, Ataki Paniki, Fobie. Grupa integracyjna	43.7
10.	Abstynenci	24.7
11.	Rzucam palenie. Grupa wsparcia	72.2
12.	Łuszczyca / Psoriasis Grupa Wsparcia	9.7
13.	Łuszczyca z innej perspektywy.	16
14.	Reumatyzm, zapalenie stawów, RZS, ŁZS, Fibromialgia, ZZSK, Dna moczanowa	24
15.	Dermatologia - walczymy o zdrową skórę	30.7
16.	Alergia - Poważni Alergicy	4.1
17.	Choroby Tarczycy ǀ Hashimoto ǀ Niedoczynność ǀ Dieta ǀ Wsparcie	34.7
18.	Nadczynność Tarczycy Gravesa-Basedowa	5.5
19.	Atopowe Zapalenie Skóry Alergia, AZS grupa wsparcia dla rodziców	48.5
20.	Lekarze, pacjenci, szpitale, ratownictwo /OCHRONA ZDROWIA - wiadomości, dyskusje	23.8
21.	Kosmetolodzy, kosmetyczki, lekarze-opinie	90.9
22.	Jak będzie w szpitalu? - sekcja medyczna - miesiąc stanu wyjątkowego	21
23.	COVID-19 - Koronawirus - grupa wsparcia dla pozytywnych i ozdrowieńców.	8.9

24.	Pielęgniarki/ Pielęgniarze - najlepszy zawód :)	69
25.	Grupa Pielęgniarki Cyfrowe	12.2
26.	Badania naukowe – organizatorzy i badani	4.1
27.	Wypełniamy ankiety - do prac dyplomowych, licencjackich, magisterskich	13
28.	Ankiety- chętnie pomogę wypełnić	24
29.	Badania, ankiety, wywiady	5.9
30.	BTW ǀ BEZ TABU WSZYSCY	103.8
31.	Medyczna marihuana w Polsce	15.3
32.	"Homeopatia Polska"- klasyczna, kliniczna. Odtruwanie Homeo. Porady online.	30.5
33.	Dieta wg Medycyny Chińskiej - Porady	13.8
34.	Zioła i Terapie Naturalne	37
35.	Naturalne metody leczenia –zioła- alternatywne metody - Wiedza o zdrowiu	62.5
36.	🌿Wylecz się Ziołami (Zioła, Naturalne metody leczenia, Porady, Natura)🌿	159
37.	Grupa Stawianie Baniek	46.2
38.	TCM Medycyna Chińska dla każdego	4.7

Table S2. STROBE Statement—Checklist.

	Item No	Recommendation	Page
**Title and abstract**	1	(*a*) Indicate the study's design with a commonly used term in the title or the abstract	1
		(*b*) Provide in the abstract an informative and balanced summary of what was done and what was found	1
**Introduction**			
Background/rationale	2	Explain the scientific background and rationale for the investigation being reported	1-2
Objectives	3	State specific objectives, including any prespecified hypotheses	2
**Methods**			
Study design	4	Present key elements of study design early in the paper	2-6
Setting	5	Describe the setting, locations, and relevant dates, including periods of recruitment, exposure, follow-up, and data collection	2-6
Participants	6	(*a*) Give the eligibility criteria, and the sources and methods of selection of participants	2-6
Variables	7	Clearly define all outcomes, exposures, predictors, potential confounders, and effect modifiers. Give diagnostic criteria, if applicable	2-6
Data sources/ measurement	8*	For each variable of interest, give sources of data and details of methods of assessment (measurement). Describe comparability of assessment methods if there is more than one group	2-6
Bias	9	Describe any efforts to address potential sources of bias	2-6
Study size	10	Explain how the study size was arrived at	2-6
Quantitative variables	11	Explain how quantitative variables were handled in the analyses. If applicable, describe which groupings were chosen and why	2-6
Statistical methods	12	(*a*) Describe all statistical methods, including those used to control for confounding	6
		(*b*) Describe any methods used to examine subgroups and interactions	N/A
		(*c*) Explain how missing data were addressed	2-6
		(*d*) If applicable, describe analytical methods taking account of sampling strategy	N/A
		(*e*) Describe any sensitivity analyses	N/A
**Results**			
Participants	13*	(a) Report numbers of individuals at each stage of study—eg. numbers potentially eligible, examined for eligibility, confirmed eligible, included in the study, completing follow-up, and analysed	7
		(b) Give reasons for non-participation at each stage	7
		(c) Consider use of a flow diagram	N/A
Descriptive data	14*	(a) Give characteristics of study participants (eg demographic, clinical, social) and information on exposures and potential confounders	7

		(b) Indicate number of participants with missing data for each variable of interest	7
Outcome data	15*	Report numbers of outcome events or summary measures	N/A
Main results	16	(*a*) Give unadjusted estimates and, if applicable, confounder-adjusted estimates and their precision (eg, 95% confidence interval). Make clear which confounders were adjusted for and why they were included	9-14
		(*b*) Report category boundaries when continuous variables were categorized	N/A
		(*c*) If relevant, consider translating estimates of relative risk into absolute risk for a meaningful time period	N/A
Other analyses	17	Report other analyses done—eg analyses of subgroups and interactions, and sensitivity analyses	N/A
**Discussion**			
Key results	18	Summarise key results with reference to study objectives	14
Limitations	19	Discuss limitations of the study, taking into account sources of potential bias or imprecision. Discuss both direction and magnitude of any potential bias	15
Interpretation	20	Give a cautious overall interpretation of results considering objectives, limitations, multiplicity of analyses, results from similar studies, and other relevant evidence	15
Generalisability	21	Discuss the generalisability (external validity) of the study results	N/A
**Other information**			
Funding	22	Give the source of funding and the role of the funders for the present study and, if applicable, for the original study on which the present article is based	16

*Give information separately for exposed and unexposed groups.

Note: An Explanation and Elaboration article discusses each checklist item and gives methodological background and published examples of transparent reporting. The STROBE checklist is best used in conjunction with this article (freely available on the Web sites of PLoS Medicine at http://www.plosmedicine.org/, Annals of Internal Medicine at http://www.annals.org/, and Epidemiology at http://www.epidem.com/). Information on the STROBE Initiative is available at www.strobe-statement.org.

International Journal of
Environmental Research and Public Health

MDPI

Table S3. The results of the agreement in the assessment of the individual items of the I-CAM-Q scale by the experts using the Delphi method.

	**Items**	**M ± SD**	**Cons.**
	Round one		
1.	Visiting health care providers:	2.2 ± 0.98	10%
†	-	-	-
1.1.	Physician	3.9 ± 0.3	90%
†	-	-	-
1.2.	Chiropractior	4 ± 0	100%
1.3.	Herbalist	3 ± 1.26	60%
1.4.	Homeopath	4 ± 0	100%
1.5.	Acupuncturist	3.9 ± 0.3	90%
1.6.	Spiritual healer	3.9 ± 0.3	90%
1.7.	*Znachor*	3.7 ± 0.46	70%
2.	Complementary treatments received from physicians:	4 ± 0	100%
2.1.	Manipulation	3.9 ± 0.3	90%
2.2.	Homeopathy	4 ± 0	100%
2.3.	Acupuncture	4 ± 0	100%
2.4.	Herbs	4 ± 0	100%
2.5.	Spiritual healing	3.8 ± 0.4	80%
2.6.	Cupping therapy	3.9 ± 0.3	90%
3.	Use of herbal medicine and dietary supplements:	4 ± 0	100%
3.1.	Herbs/ Herbal medicine	3.9 ± 0.3	90%
3.2.	Vitamins/minerals	4 ± 0	100%
3.3.	Homeopathic remedies	2.4 ± 1.2	30%
3.4.	Other Supplements	4 ± 0	100%
4.	Self help practices:	3.9 ± 0.3	90%
4.1.	Meditation	4 ± 0	100%
4.2.	Yoga	4 ± 0	100%
4.3.	Quigong	3.9 ± 0.3	90%
4.4.	Tai Chi	3.9 ± 0.3	90%
4.5.	Relaxation techniques	4 ± 0	100%
4.6.	Visualization	3.9 ± 0.3	90%
4.7.	Attended traditional healing ceremony	3.9 ± 0.3	90%
4.8.	Praying for own health	4 ± 0	100%
	Round two		
1.	Visiting practitioners of complementary and alternative medicine:	3.9 ± 0.3	90%
†	*Medical practitioners of complementary and alternative medicine*	4 ± 0	100%
1.1.	Physician	3.9 ± 0.3	90%
†	*Non-medical practitioners of complementary and alternative medicine*	3.9 ± 0.3	90%

1.2.	Chiropractor	4 ± 0	100%
1.3.	Herbalist/ Phytotherapist	4 ± 0	100%
1.4.	Homeopath	4 ± 0	100%
1.5.	Acupuncturist	3.9 ± 0.3	90%
1.6.	Spiritual healer	3.9 ± 0.3	90%
1.7.	*Znachor/ Szeptucha*	4 ± 0	100%
2.	Complementary treatments received from physicians:	4 ± 0	100%
2.1.	Manipulation	3.9 ± 0.3	90%
2.2.	Homeopathy	4 ± 0	100%
2.3.	Acupuncture	3.9 ± 0.3	90%
2.4.	Herbs	4 ± 0	100%
2.5.	Spiritual healing	3.9 ± 0.3	90%
2.6.	Cupping therapy	3.9 ± 0.3	90%
3.	Use of herbal medicine and dietary supplements:	4 ± 0	100%
3.1.	Herbs/ herbal medicine	3.9 ± 0.3	90%
3.2.	Vitamins/ minerals	4 ± 0	100%
3.3.	Homeopathic medicines	4 ± 0	100%
3.4.	Other supplements	4 ± 0	100%
4.	Self help practices:	3.9 ± 0.3	90%
4.1.	Meditation	4 ± 0	100%
4.2.	Yoga	4 ± 0	100%
4.3.	Quigong	3.9 ± 0.3	90%
4.4.	Tai Chi	3.9 ± 0.3	90%
4.5.	Relaxation techniques	4 ± 0	100%
4.6.	Visualization	3.9 ± 0.3	90%
4.7.	Attended traditional healing ceremony	3.9 ± 0.3	90%
4.8.	Praying for own health	4 ± 0	100%

M: mean; SD: standard deviation; cons.: consensus obtained.
† - Items added in the second round

Table S4. The frequency, motivation and evaluation of herbal medicine and dietary supplements use in the study group during the last 12 months. 70

Use of herbal medicine and dietary supplements:	Used [a,#]	Motivation [a,##]				Missing data	Usefulness of the product [a,##]				
		Chronic disease	Acute illness	To improve well-being	Others		Very	Some what	No at all	Do not know	Missing data
Herbs / Herbal Medicine:											
Mixtures of herbs	94 (9.4)	47 (50.0)	13 (13.8)	20 (21.3)	13 (13.8)	1 (1.1)	61 (64.9)	19 (20.2)	1 (1.1)	10 (10.6)	3 (3.2)
Herbal dietary supplements	59 (5.9)	26 (44.1)	5 (8.5)	20 (33.9)	8 (13.6)	0	39 (66.1)	12 (20.3)	2 (3.4)	5 (8.5)	1 (1.7)
Herbal medicinal products	39 (3.9)	10 (25.6)	12 (30.8)	10 (25.6)	5 (12.8)	2 (5.1)	25 (64.1)	10 (25.6)	0	2 (5.1)	2 (5.1)
Mixtures of Chinese herbs	30 (3.0)	15 (50.0)	4 (13.3)	11 (36.7)	0	0	16 (53.3)	14 (46.7)	0	0	0
Nettle	38 (3.8)	19 (50.0)	5 (13.2)	11 (28.9)	2 (5.3)	1 (2.6)	30 (78.9)	6 (15.8)	0	1 (2.6)	1 (2.6)
Lemon balm	34 (3.4)	3 (8.8)	4 (11.8)	25 (73.5)	1 (2.9)	1 (2.9)	16 (47.1)	13 (38.2)	4 (11.8)	0	1 (2.9)
Ashwagandha	27 (2.7)	8 (29.6)	0	16 (59.3)	1 (3.7)	2 (7.4)	14 (51.9)	10 (37.0)	0	1 (3.7)	2 (7.4)
Mint	26 (2.6)	6 (23.1)	7 (26.9)	12 (46.2)	1 (3.8)	0	13 (50.0)	10 (38.5)	0	3 (11.5)	0
Milk Thistle	22 (2.2)	11 (50.0)	4 (18.2)	6 (27.3)	1 (4.5)	0	14 (63.6)	5 (22.7)	0	3 (13.6)	0
Dandelion	21 (2.1)	5 (23.8)	5 (23.8)	7 (33.3)	3 (14.3)	1 (4.8)	19 (90.5)	2 (9.5)	0	0	0
CBD oil*	21 (2.1)	13 (61.9)	0	8 (38.1)	0	0	16 (76.2)	5 (23.8)	0	0	0
Salvia	16 (1.6)	2 (12.5)	9 (65.3)	4 (25.0)	1 (6.3)	0	10 (62.5)	6 (37.5)	0	0	0
Cannabis	16 (1.6)	8 (50.0)	4 (25.0)	4 (25.0)	0	0	13 (81.3)	3 (18.8)	0	0	0
St. John's wort	15 (1.5)	5 (33.3)	2 (13.3)	7 (46.7)	1 (6.7)	0	13 (86.7)	2 (13.3)	0	0	0
Black Seed	15 (1.5)	8 (53.3)	4 (26.7)	3 (20.0)	0	0	9 (60.0)	4 (26.7)	1 (6.7)	1 (6.7)	0
Equisetum	14 (1.4)	4 (28.6)	1 (7.1)	4 (28.6)	5 (35.7)	0	14 (100)	0	0	0	0
Chamomile	12 (1.2)	4 (33.3)	1 (8.3)	7 (58.3)	0	0	5 (41.7)	7 (58.3)	0	0	0
Cistus	11 (1.1)	3 (27.3)	0	4 (36.4)	4 (36.4)	0	6 (54.5)	3 (27.3)	1 (9.1)	1 (9.1)	0

Cleavers	11 (1.1)	3 (27.3)	1 (9.1)	4 (36.4)	2 (18.2)	1 (9.1)	7 (63.6)	4 (36.4)	0	0	0
Elderberry	11 (1.1)	0	4 (36.4)	4 (36.4)	3 (27.3)	0	11 (100)	0	0	0	0
Bee products	9 (0.9)	4 (44.4)	3 (33.3)	2 (22.2)	0	0	6 (66.7)	3 (33.3)	0	0	0
Medicinal mushrooms	8 (0.8)	6 (75.0)	0	1 (12.5)	1 (12.5)	0	4 (50.0)	2 (25.0)	0	2 (25.0)	0
Other herbs	325 (32.5)	153 (47.1)	49 (15.1)	88 (27.1)	30 (9.2)	5 (1.5)	225 (69.2)	77 (23.7)	6 (1.8)	13 (4.0)	4 (1.2)
Unspecified by the respondent	66 (6.6)	22 (33.3)	4 (6.1)	24 (36.4)	3 (4.5)	13 (19.7)	35 (53.0)	14 (21.2)	3 (4.5)	2 (3.0)	12 (18.2)
Not herb/plant	60 (6.0)	29 (48.3)	1 (1.7)	22 (36.7)	5 (8.3)	3 (5.0)	44 (73.3)	13 (21.7)	0	0	3 (5.0)
Vitamins / minerals:											
ADEK complex	30 (2.9)	11 (36.7)	0 (0)	10 (33.3)	9(30.3)	0 (0)	27 (90.0)	2 (6.7)	0	1 (3.3)	0
Vitamin D3	210 (20.0)	69 (32.9)	3 (1.4)	87 (41.4)	46 (21.9)	5 (2.4)	121 (57.6)	16 (7.6)	1 (0.5)	35 (16.7)	37 (17.6)
Vitamin D3 + K2	48 (4.6)	20 (41.7)	0	20 (41.9)	7 (14.6)	1 (2.1)	40 (83.3)	2 (4.2)	0	3 (6.3)	3 (6.3)
Vitamin C	124 (11.8)	33 (26.6)	12 (9.7)	52 (41.9)	15 (12.1)	12 (9.7)	94 (75.8)	9 (7.3)	0	7 (5.6)	14 (11.3)
Vitamin B complex	68 (6.5)	34 (50.0)	0	30 (44.1)	3 (4.4)	1 (1.5)	44 (64.7)	7 (10.3)	0	12 (17.6)	5 (7.4)
Vitamin B12	32 (3.0)	12 (13.7)	1 (3.1)	10 (31.3)	8 (25.0)	1 (3.1)	22 (68.8)	2 (6.3)	0	5 (15.6)	3 (9.4)
Vitamin B9	13 (1.2)	4 (30.8)	1 (7.7)	4 (30.8)	4 (30.8)	0	7 (53.8)	2 (15.4)	0	4 (30.8)	0
Mulitivitamins	33 (3.1)	6 (18.2)	1 (3.0)	19 (57.6)	7 (21.2)	0	21 (63.6)	2 (6.1)	0	3 (9.1)	7 (21.2)
Specific multivitamins	36 (3.4)	11 (30.6)	1 (2.8)	11 (30.6)	12 (33.3)	1 (2.8)	13 (36.1)	7 (19.4)	0	11 (30.6)	5 (13.9)
Magnesium	123 (11.7)	45 (36.6)	2 (1.6)	55 (44.7)	14 (11.4)	7 (5.7)	76 (61.8)	18 (14.6)	0	8 (6.5)	21 (17.1)
Zinc	54 (5.1)	24 (44.4)	3 (5.6)	16 (29.6)	9 (16.7)	2 (3.7)	34 (63.0)	11 (20.4)	1 (1.9)	5 (9.3)	3 (5.6)
Potassium	16 (1.5)	9 (56.3)	1 (6.3)	3 (18.8)	3 (18.8)	0	8 (50.0)	5 (31.3)	0	1 (6.3)	2 (12.5)
Selenium	19 (1.8)	10 (52.6)	0	8 (42.1)	1 (5.3)	0	10 (52.6)	6 (31.6)	0	2 (10.5)	1 (5.3)
Iron	13 (1.2)	9 (69.2)	2 (15.4)	1 (7.7)	0	1 (7.7)	7 (53.8)	2 (15.4)	1 (7.7)	2 (15.4)	1 (7.7)
Iodine	12 (1.1)	5 (41.7)	0	3 (25.0)	4 (33.3)	0	11 (91.7)	0	0	1 (8.3)	0
Omega fatty acids	21 (2.0)	7 (33.3)	0	10 (47.6)	4 (19.0)	0	13 (61.9)	4 (19.0)	0	2 (9.5)	2 (9.5)
Other vitamins/minerals	177 (16.8)	86 (48.6)	8 (4.5)	56 (31.6)	19 (10.7)	8 (4.5)	111 (62.7)	27 (15.3)	1 (0.6)	21 (11.9)	17 (9.6)

Unspecified by the respondent	23 (2.2)	5 (21.7)	0	14 (60.9)	4 (17.4)	0	18 (78.3)	1 (4.3)	1 (4.3)	1(4.3)	2 (8.7)

Date are presented as: [a] n(%);[#] percentages are calculated in relation to the total sample population; [##] percentages are calculated in relation to the number of people using a given health care provider;
